# Effect of probiotic supplementation combined with bismuth-containing quadruple therapy on gut microbiota during *Helicobacter pylori* eradication: a randomized, double-blind, placebo-controlled trial

**DOI:** 10.3389/fnut.2024.1484646

**Published:** 2024-10-16

**Authors:** Zhongxue Han, Yueyue Li, Xueping Nan, Tao Zhou, Lixiang Li, Yanqing Li

**Affiliations:** ^1^Department of Gastroenterology, Qilu Hospital of Shandong University, Jinan, China; ^2^Shandong Provincial Clinical Research Center for Digestive Disease, Jinan, China; ^3^Department of Geriatric Medicine, Qilu Hospital of Shandong University, Jinan, China; ^4^Key Laboratory of Cardiovascular Proteomics of Shandong Province, Qilu Hospital of Shandong University, Jinan, China

**Keywords:** *Helicobacter pylori* eradication, probiotics, gut microbiota, 16S rRNA gene sequence, bismuth quadruple therapy

## Abstract

**Background:**

*Helicobacter pylori* (*H. pylori*) eradication has been reported to affect gut microbiota distribution. This study aimed to evaluate the effect of probiotic supplementation on the gastrointestinal microbiota during eradication and the efficacy of bismuth-containing quadruple therapy.

**Methods:**

One hundred treatment-naïve *H. pylori*-positive patients were randomly assigned 1:1 to receive 14-day bismuth-containing quadruple therapy (esomeprazole, bismuth, amoxicillin, and clarithromycin) combined with the probiotic (*Bifidobacterium animalism* subsp. *lactis BLa80*) or placebo. The Gastrointestinal Symptom Rating Scale (GSRS) was completed before and after treatment. Stool samples were collected for 16S rRNA gene sequencing at weeks 0, 2, and 10.

**Results:**

No significant difference in the eradication rate was observed between the two groups. The incidence of adverse events, especially nausea (*p* = 0.029), was lower in the probiotic group. After treatment, the GSRS score decreased significantly in the probiotic group (*p* = 0.039). The gut microbiota underwent considerable changes immediately following eradication treatment, predominantly characterized by an increase in Proteobacteria at the expense of commensal Firmicutes and Bacteroidota, but gradually returned to baseline after eight weeks. By week 10, beneficial genera such as *Lachnoclostridium*, *Parasutterella*, *Hungatella*, and *Akkermansia* were notably enriched in the probiotic group. Additionally, the correlation networks in the probiotic group were closer to their initial levels at week 10 compared to the placebo group.

**Conclusion:**

Disturbances in the gut microbiota following *H. pylori* treatment appeared to be temporary, and probiotic supplementation could mitigate antibiotic-induced alterations in the gut microbiota. This study also provided evidence supporting the effectiveness of probiotics in alleviating gastrointestinal symptoms.

## Introduction

*Helicobacter pylori* (*H. pylori*) is one of the most prominent causes of gastric cancer, infecting half of the global population, and causing a heavy health burden (1). *H. pylori* eradication has been confirmed to significantly reduce morbidity and mortality from gastric cancer (2). Guidelines recommend bismuth-containing quadruple therapy as first-line treatment, and all *H. pylori*-positive patients should receive eradication therapy according to the Kyoto Global Consensus report (3, 4). However, the widespread use of antibiotics at the population level has raised concerns regarding the emergence of antibiotic resistance and gut microbiota distribution, which can lead to a vicious cycle of declining eradication rate (5). Large-scale *H. pylori* eradication remains a great challenge.

Gut microbiota plays a significant role in homeostasis, crucial for maintaining the gastrointestinal microecological environment (6). *H. pylori* eradication using antibiotics can disrupt the gastrointestinal microbial community, increase the expression of antibiotic resistance genes, and select drug-resistant species from the gut microbiota; further decreasing the eradication rate (7). Furthermore, gut dysbiosis is associated with adverse effects, such as diarrhea, nausea, and vomiting, which influence drug compliance (8). Alterations in the gut microbiota may persist for several months, and *Clostridium difficile* infection and pseudomembranous colitis have been reported associated with *H. pylori* eradication (9). Therefore, effective measures are needed to alleviate the profound effects of the drugs involved in *H. pylori* treatment on the gut microbiota.

Probiotics are live microorganisms that are beneficial to host health when administered in adequate amounts (10). They can mediate health benefits through alterations in gut microbiota composition, production of antibacterial substances, and immunological regulation (10). Probiotic supplementation may be an option to relieve gut dysbiosis during *H. pylori* antibiotic treatment. Several studies have shown that the addition of probiotics mitigates the side effects of *H. pylori* eradication, improves patient compliance, and even increases the eradication rate; however, the results are inconsistent (11). Whether probiotic administration can reduce antibiotic-associated gut microbiota imbalance and maintain a stable microbial ecosystem remains under investigation. Elucidating the effect of probiotic supplementation on the gut microbiota during *H. pylori* eradication is invaluable to clinicians and patients.

This randomized, double-blind, placebo-controlled study aimed to evaluate the impact of *H. pylori* eradication on the gut microbiota, and determine whether concomitant probiotic therapy could relieve gut microbiota disturbance, reduce side effects, and improve the eradication rate.

## Materials and methods

### Participants

One hundred consecutive *H. pylori* infected patients were recruited at Qilu Hospital of Shandong University in Shandong Province between April 2023 and August 2023 under the following inclusion criteria: (1) 18–70 years old; (2) diagnosed with *H. pylori* infection by urea breath test (UBT) or rapid urease test (RUT). Exclusion criteria were as follows: (1) previous history of *H. pylori* eradication; (2) know allergy to drugs prescribed in this study or any contraindication to the eradication therapy; (3) pregnant or lactating women; (4) administration of antibiotics, probiotics, acid-suppressing drugs or any other drugs which could influence the gut microbiota 3 months before; (5) previous history of gastrointestinal surgery; (6) severe concurrent diseases; (7) alcohol or drug abusers; (8) active gastrointestinal diseases such as peptic ulcer, inflammatory bowel disease, and celiac disease; (9) chronic diarrhea or constipation; (10) subjects who could not provide informed consent.

### Study design

This prospective, single-center, randomized, double-blind, placebo-controlled trial was approved by the Medical Ethics Committee of Qilu Hospital of Shandong University (approval number: KYLL-202210-011), and adhered to the principles of Good Clinical Practice and the Declaration of Helsinki. All the patients were fully informed of the possible benefits and potential risks of participating in this trial, and voluntarily signed the written informed consent. It has been registered at the ClinicalTrials.gov (accession ID: NCT05662514). The trial was reported in accordance with the CONSORT statement.

Eligible patients were randomly assigned to the probiotic group or the placebo group in a 1:1 ratio. Randomization was conducted by a random number sequence generated by SAS® 9.4 (SAS Institute Inc., Cary, NC, USA). Patients and clinical researchers were blinded to the randomization and study products. Patients in both groups were treated by bismuth-containing quadruple therapy (esomeprazole 20 mg, amoxicillin 1,000 mg, clarithromycin 500 mg, bismuth potassium citrate 220 mg, dosed morning, and evening) for two weeks. The probiotic group was supplemented with the probiotic (*Bifidobacterium animalism* subsp. *lactis* BLa80) and the placebo group was supplemented with the placebo, one packet once daily in the evening for two weeks concomitant with bismuth-containing quadruple therapy. The interval between antibiotics and the probiotic/placebo was at least 2 h.

The investigational probiotic and placebo involved in the study were both manufactured and funded by Wecare Probiotics Co., Ltd., Jiangsu, P.R. China. The probiotic formulation included maltodextrin and a single strain *Bifidobacterium animalism* subsp. *lactis* BLa80 at a concentration of 1*10^11^ colony-forming units (CFU) per dose, whereas the placebo consisted solely of maltodextrin. To ensure the double-blinding, the probiotic and placebo were prepared in indistinguishable powder form and packed as 14 packets (3 g each packet) in individual boxes. The boxes should be kept in a dry place below 30°C and away from direct sunlight.

### Procedures

**Figure 1:**
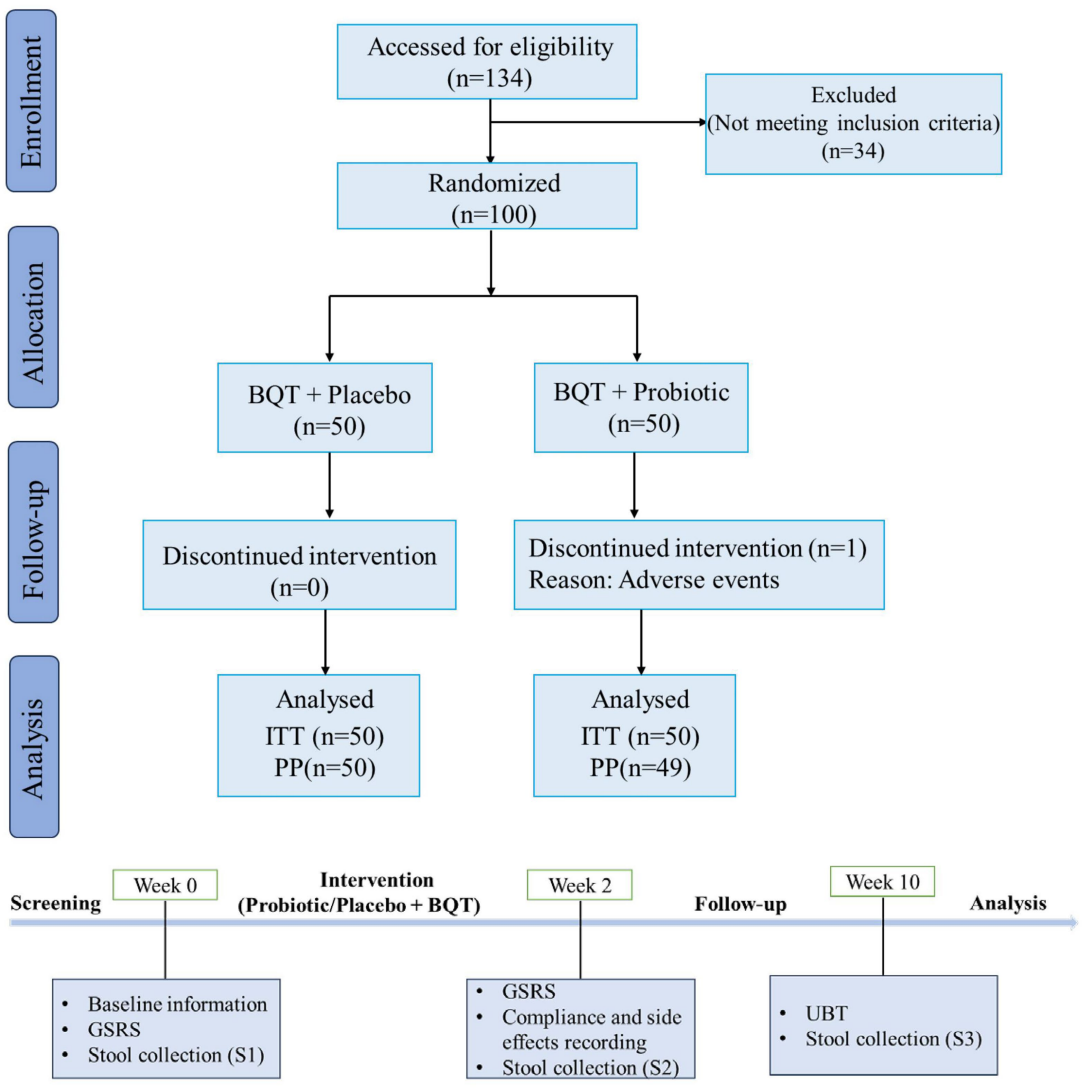
Flow diagram showing the study design. BQT, bismuth quadruple therapy; ITT, intention-to-treat; PP, per protocol; GSRS, Gastrointestinal Symptom Rating Scale; UBT, Urea Breath Test.

Eligible patients were identified in the screening period (day-14 to day −1, week 0) and baseline demographic data were collected. They were also asked to answer a Gastrointestinal Symptom Rating Scale (GSRS) questionnaire (12). Then, subjects received eradication treatment (day 1 to day 14, week 2) and answered the GSRS questionnaire again at the end of this period. Compliance and adverse effects were evaluated by means of patient’s diary and product account ability. Eight weeks after treatment (week 10), patients were followed up by ^13^C-UBT to ascertain the infection status. All patients were forbidden to take other antibiotics and probiotics that were not included in this trial. A flow diagram of this trial was shown in Figure 1.

### Outcomes

The primary outcome was the changes of gut microbes during *H. pylori* eradication. The secondary outcomes were *H. pylori* eradication rates, assessed by intention-to-treat (ITT) and per-protocol (PP) analyses, the frequencies of adverse effects, the GSRS scores, and compliance to the therapy.

### Stool collection

Three stool samples were collected before initiation of eradication (week 0), immediately after completion of treatment (week 2), and 8 weeks after eradication (week 10) by disposable sterile feces collection tubes. Samples were immediately frozen at −80°C.

### DNA extraction, PCR amplification and sequencing

Total microbial genomic DNA was extracted from stool samples via the E.Z.N.A.^®^ soil DNA Kit (Omega Bio-tek, Norcross, GA, United States). The quality and concentration of DNA were measured via 1.0% agarose gel electrophoresis as well as a NanoDrop^®^ ND-2000 spectrophotometer (Thermo Scientific Inc., United States) and then them were stored at −80°C. The V3-V4 region of the bacterial 16S rRNA gene were amplified with the primers named 338F (5’-ACTCCTACGGGAGGCAGCAG-3′) and 806R (5’-GGACTACHVGGGTWTCTAAT-3′) using an ABI GeneAmp^®^ 9,700 PCR thermocycler (ABI, CA, United States) (13). All samples were amplified in triplicate. Then the PCR product was extracted from 2% agarose gel and purified using the AxyPrep DNA Gel Extraction Kit (Axygen Biosciences, Union City, CA, United States). After that, the purified PCR products were further quantified using Quantus^™^ Fluorometer (Promega, United States) and sequenced by the Illumina MiSeq PE300 platform (Illumina, San Diego, United States) by Majorbio Bio-Pharm Technology Co. Ltd. (Shanghai, China).

### Data processing and analysis

After demultiplexing, the resulting sequences were quality filtered with fastp (0.19.6) (14) and merged with FLASH (v1.2.11) (15). Then the high-quality sequences were de-noised using DADA2 (16) plugin in the Qiime2 (17) (version 2020.2) pipeline with recommended parameters to obtain single nucleotide resolution. The denoised sequences are named amplicon sequence variants (ASVs). To minimize the effects of sequencing depth on alpha and beta diversity measure, the number of sequences from each sample was rarefied to 20,743, which still yielded an average Good’s coverage of 98.89%. Taxonomic assignment of ASVs was performed using the Naive bayes consensus taxonomy classifier implemented in Qiime2 based on the SILVA 16S rRNA database (v138).

Bioinformatic analysis of the gut microbiota was carried out on the Majorbio Cloud platform.^1^ Based on the ASVs information, alpha diversity indices including Chao1 richness, Shannon index, Ace index, and Simpson index were calculated with Mothur v1.30.1 (18). The similarity among the microbial communities in different fecal samples was determined by principal coordinate analysis (PCoA) via Vegan v2.5–3 package based on Bray–curtis dissimilarity. The linear discriminant analysis (LDA) effect size (LEfSe) was carried out to identify the significantly abundant taxa (phylum to genera) of bacteria among the different groups (LDA score > 2, *p* < 0.05) (19). The co-occurrence networks were constructed to explore the internal community relationships across the samples (20).

### Statistical analysis

Statistical analyses were performed using the software SPSS (IBM, Armonk, NY). Continuous data that conformed to a normal distribution were expressed as mean ± standard deviation and continuous data not following a normal distribution are presented as median and interquartile range. Categorical data were described in percentage. Eradication efficacy was performed on an ITT population where patients who dropped out were considered as treatment failures. Secondary PP analyses were performed which excluded patients lost to follow-up or prematurely withdrew before completion of the study. Multivariate regression analysis was performed to identify baseline characteristics differences between groups. Between-group comparisons of GSRS scores were using Mann–Whitney U test and within-group comparisons of GSRS scores between pre- and post-treatment were using Wilcoxon signed-rank test. All statistical tests were two-tailed. Comparisons of eradication rates and adverse effect rates between groups were using χ^2^ test or Fisher’s exact test. *p* values less than 0.05 were considered significant. Besides, a post-hoc power analysis was conducted to judge the reliability of results.

## Results

### Baseline characteristics

A total of 100 patients were randomly assigned to receive the placebo (*n* = 50) or probiotic (*n* = 50) combined with bismuth-containing quadruple therapy. During the study, one patient in the probiotic group failed to complete treatment due to allergy and was excluded from the PP analysis. The baseline characteristics of enrolled patients are presented in Table 1. No significant differences in age, gender, body mass index, smoking habits, alcohol consumption, marital status, and family history were found between the two groups. Before the eradication therapy, there was no significant difference in overall GSRS scores between the two groups (*p* = 0.920).

**Table 1:** Demographics and clinical characteristics of patients included in the study.

Characteristic	Placebo group	Probiotic group	*P* value
	(*n* = 50)	(*n* = 50)	
Age (years), mean (IQR)	35.48 (29.50–41.00)	38.26 (29.00–45.50)	0.619
Sex			
Male, *n* (%)	20 (40.00%)	20 (40.00%)	0.340
BMI, mean ± SD	23.24 ± 3.41	22.38 ± 2.60	0.131
Marital status			
Unmarried, *n* (%)	9 (18.00%)	14 (28.00%)	0.119
Family history of gastric cancer			
First-degree relatives, *n* (%)	2 (4.00%)	2 (4.00%)	0.674
Smoking, *n* (%)	8 (16.00%)	4 (8.00%)	0.994
Alcohol, *n* (%)	12 (24.00%)	8 (16.00%)	0.721
Hypertension, *n* (%)	4 (8.00%)	6 (12.00%)	0.994
Diabetes, *n* (%)	5 (10.00%)	4 (8.00%)	1.000
Education			
Illiteracy, *n* (%)	3 (6.00%)	1 (2.00%)	_
Elementary school, *n* (%)	4 (8.00%)	6 (12.00%)	0.569
High school, *n* (%)	18 (36.00%)	21 (42.00%)	0.751
College or above, *n* (%)	25 (50.00%)	22 (44.00%)	0.863

IQR, interquartile range; SD, standard deviation; BMI, body mass index.

### *Helicobacter pylori* eradication rate

As shown in Table 2, ITT analysis indicated that the eradication rates were 86.00% for the placebo group and 86.00% for the probiotic group (*p* = 1.000). PP analysis demonstrated that the eradication rates were 86.00% for the placebo group and 87.75% for the probiotic group (*p* = 0.796). Both ITT and PP analyses showed no statistically significant difference in the eradication rates between two groups.

**Table 2:** Eradication rates of *H. pylori* infection.

Group	Eradication rate, % (*n*)	
	ITT analysis	PP analysis
Placebo group	86.00% (43/50)	86.00% (43/50)
Probiotic group	86.00% (43/50)	87.76% (43/49)
*P* value	1.000	0.796

ITT, intention-to-treat; PP, per-protocol.

### Adverse events and compliance

The adverse events were summarized in Supplementary Table S1, including dysgeusia, nausea, diarrhea, vomiting, abdominal pain, bloating, constipation, and lethargy. The overall incidence of adverse events was higher in the placebo group (46.00%) than that in the probiotic group (36.00%), although with no significant difference (*p* = 0.309) due to small sample sizes. The most adverse event experienced by patients was dysgeusia in both groups.

The nausea rate of the placebo group (24.00%) was significantly higher than that in the probiotic group (8.00%) (*p* = 0.029). Meanwhile, the supplement of probiotics tended to decrease the rate of diarrhea (*p* = 0.065). The compliance rates of the placebo group and the probiotics group were 100.00 and 98.00%, respectively (*p* = 1.000).

### GSRS scores

The GSRS scores were summarized in Supplementary Table S2 and visualized in Supplementary Figure S1. Compared with pretreatment GSRS scores, a significant reduction of post-treatment GSRS scores in both two groups by a within-group analysis (*p* < 0.001). A between-group analysis showed a significantly greater score reduction in the probiotic group than that in the placebo group (*p* = 0.041).

### The diversity of the gut microbiota changed during *Helicobacter pylori* eradication

**Figure 2:**
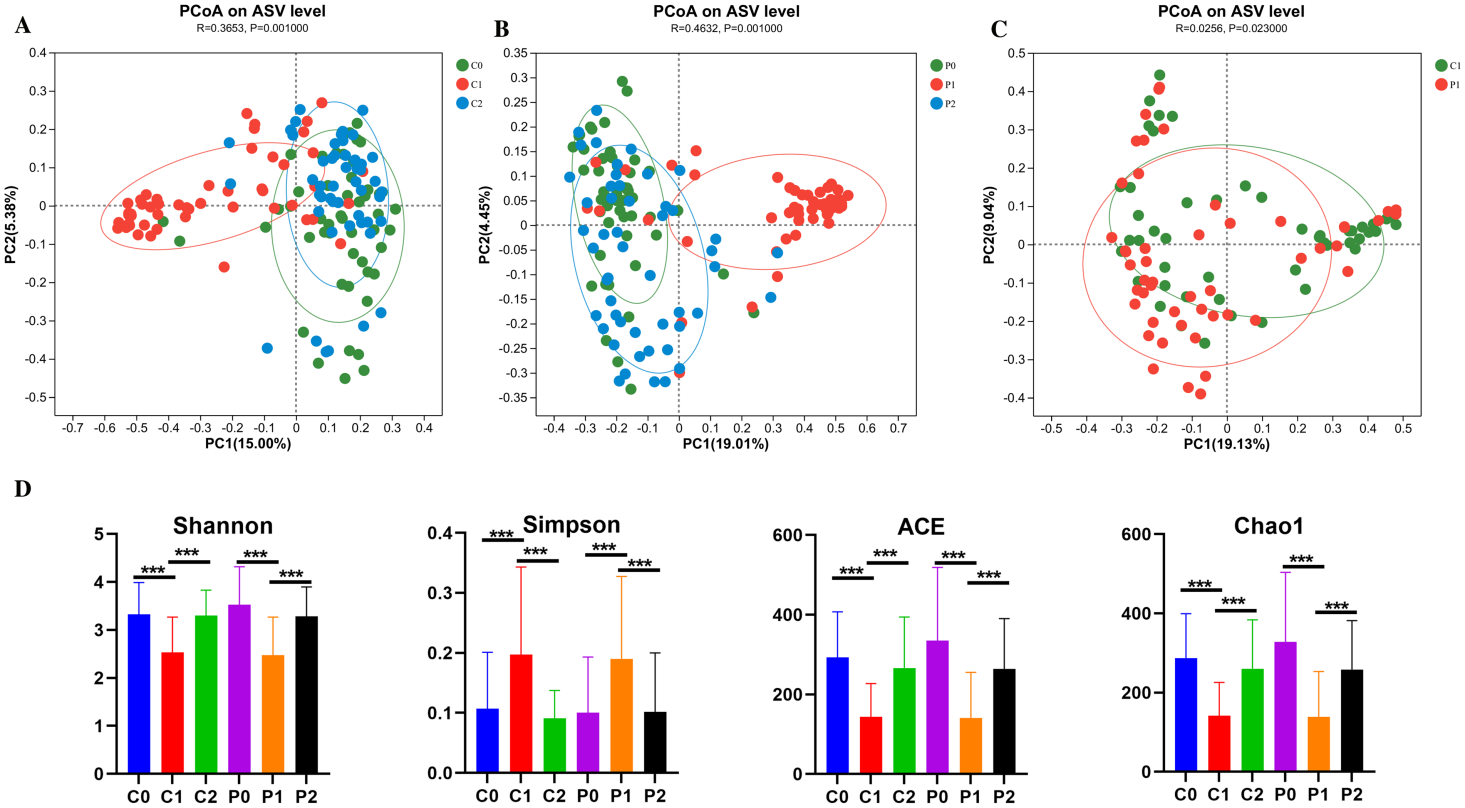
The diversity of the gut microbiota changed during *Helicobacter pylori* eradication. **(A)** PCoA on ASV level in the placebo group. **(B)** PCoA on ASV level in the probiotic group. **(C)** PCoA on ASV level compared between the placebo and probiotic group in week 2. **(D)** α-diversity of microbiota. PCoA, principal coordinate analysis; ASV, amplicon sequence variant; P, probiotic group; C, placebo group; P0 (*n* = 50)/C0 (*n* = 50), pretreatment; P1 (*n* = 50)/C1 (*n* = 50), cessation of quadruple therapy; P2 (*n* = 50)/C2 (*n* = 50), 8 weeks after quadruple therapy; *, *p* < 0.05; **, *p* < 0.01, ***, *p* < 0.001.

Antibiotics have an impact on the gut microbiota, while probiotics also have an impact on the microbiota. Therefore, total 300 fecal samples were collected from two groups at different time points for high-throughput sequencing of microbial communities to study changes in the gut microbiota. The *β*-diversity of microbiota in the probiotic group and the placebo group was similar before *H. pylori* eradication (R = −0.0081, *p* = 0.781) (Supplementary Figure S2A). As shown in Figures 2A,B, the *β*-diversity of microbiota changed significantly from day-1 to week 2, and then to week 10 both in the placebo group (R = 0.3653, *p* = 0.001) and the probiotic group (R = 0.4632, *p* = 0.001) based on PCoA. At week 2, it was found that the *β*-diversity of microbiota of the probiotic group changed significantly from that of the placebo group (R = 0.0256, *p* = 0.023) (Figure 2C). But this difference disappeared at week 10 (R = −0.0144, *p* = 0.924) (Supplementary Figure S2D). Meanwhile, we also found that the β-diversity of microbiota of the placebo group returns to its initial level at week 10 (R = 0.017, *p* = 0.086) (Supplementary Figure S2B). However, the β-diversity of microbiota of the probiotic group at week 10 was still different with that of day −1 (R = 0.0599, *p* = 0.001) (Supplementary Figure S2C).

The *α*-diversity of microbiota was also compared and the results were shown in Figure 2D. It was found that the community diversity decreased significantly after *H. pylori* eradication at week 2 based on the Shannon and Simpson index in both the probiotic group (*p* = 0.001) and the placebo group (*p* = 0.001). And then, the community diversity increased significantly to the initial level at week 10 in both the probiotic group (*p* = 0.001) and the placebo group (*p* = 0.001). The trend of changes in community richness based on Chao1 and Ace is also the same with the community diversity.

### Changes in microbial community composition during *Helicobacter pylori* eradication

**Figure 3:**
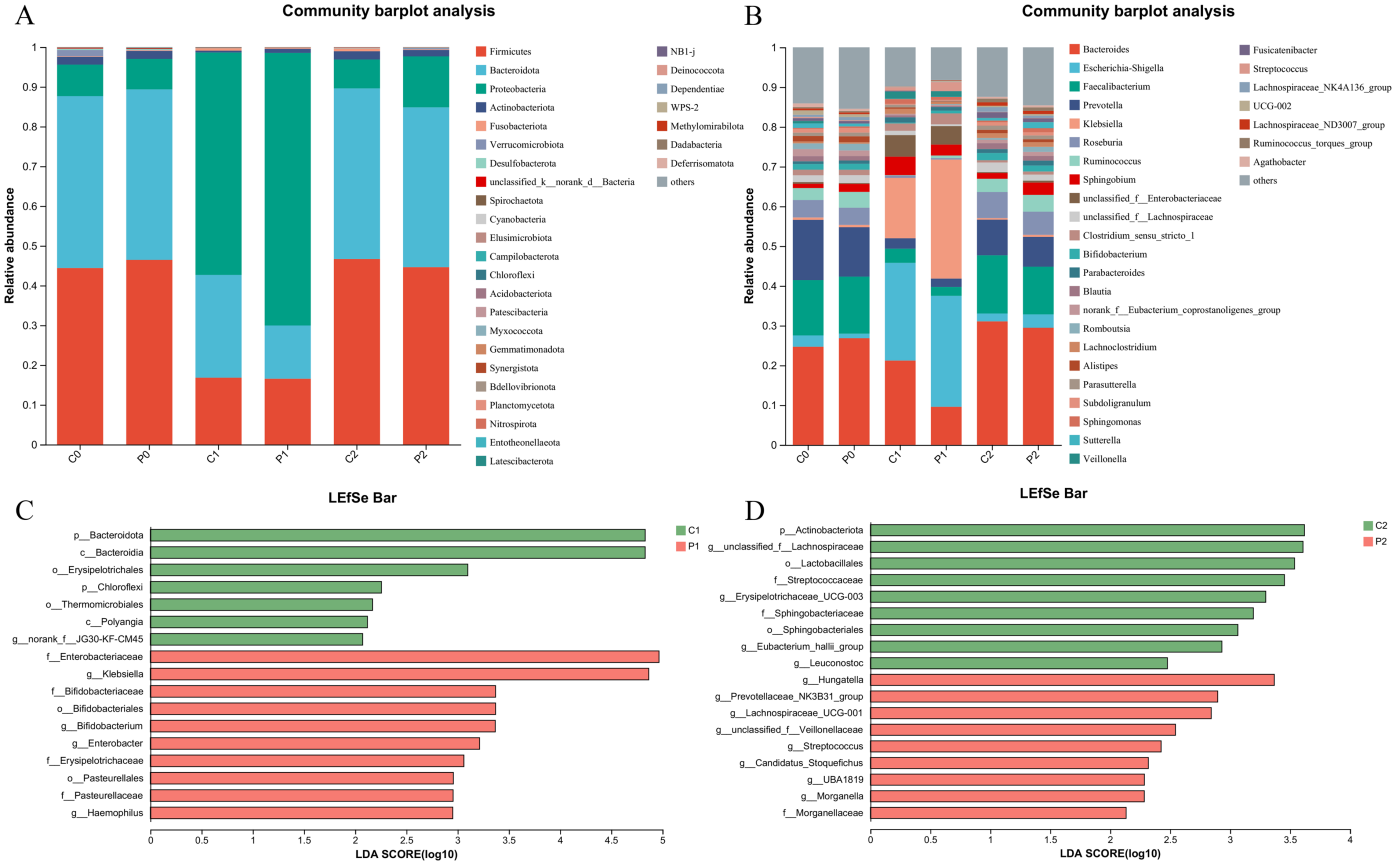
Changes in microbial community composition during Helicobacter pylori eradication. **(A)** The community composition of the gut microbiota at phylum level. **(B)** The community composition of the gut microbiota at genus level. **(C)** Changes of the gut microbiota using LEfSe analysis in week 2. **(D)** Changes of the gut microbiota using LEfSe analysis in week 10. P, probiotic group; C, placebo group; P0 (*n* = 50)/C0 (*n* = 50), pretreatment; P1 (*n* = 50)/C1 (*n* = 50), cessation of quadruple therapy; P2 (*n* = 50)/C2 (*n* = 50), 8  weeks after quadruple therapy; LDA, linear discriminant analysis.

The community composition of the gut microbiota was obtained at day-1, week 2 and week 10 as shown in Figure 3. At phylum level, the microbe of Firmicutes and Bacteroidota decreased at week 2. After discontinuation of antibiotics, their proportion returns to the initial level. On the contrast, the microbe of Proteobacteria increased at week 2 and it also returns to the initial level at week 10 (Figure 3A). At genus level, the microbe of *Bacteroides*, *Faecalibacterium*, *Prevotella*, *Roseburia*, and *Ruminococcus* decreased as well as genus of *Escherichia-Shigella*, and *Klebsiella* increased at week 2. After discontinuation of antibiotics, their proportion was close to returning to the initial level (Figure 3B).

We further investigated the changes of the gut microbiota using LEfSe analysis. When compared the gut microbiota of the probiotic group and the placebo group at the same time point, it was found that genera of *Klebsiella*, *Bifidobacterium*, *Enterobacter* and *Haemopilus* enriched in the probiotic group at week 2. While, the phylum of Bacteroidota, and genus of norank_f_JG30-KF-CM45 enriched in the placebo group at week 2 (Figure 3C). Of them, the percentage of g_*Bifidobacterium* in the gut microbiota decreased after quadruple therapy from 1.41 ± 2.47 and 1.49 ± 2.41 to 0.21 ± 0.57 and 0.69 ± 2.76 in the placebo group and probiotic group, respectively. And the g_*Bifidobacterium* in the probiotic group was significantly higher than that of the placebo group as show in Supplementary Figure S3 (*p* < 0.01). At week 10, genera of g_unclasssified_f_*Lachnospiraceae*, *Erysipelotrichaceae*_UCG-003, g_*Eubacterium*_*hallii_*group and *Leuconostoc* enriched in the placebo group. Meanwhile, the genera of *Hungatella*, *Prevotellaceae*_NK3B31_group, *Lachnospiraceae*_UCG-001, and *Streptococcus* enriched in the probiotic group (Figure 3D). When compared the microbiota of the same group at different time point, it was found that genera of *Escherichia-Shigella*, *Klebsiella, Paracoccus*, *Staphylococcus*, *Herbaspirillum*, *Pseudomonas*, *etc* enriched at week 2, while genera of *Bacteroides*, g_unclasssified_f_*Lachnospiraceae*, *Blautia*, *Flavonifractor* enriched at week 10 in the placebo group (Supplementary Figure S4A). In the probiotic group, it was found that genus of *Klebsiella*, *Escherichia-Shigella*, *Veillonella*, and *Granulicatella* enrich at week 2, while genus of *Lachnoclostridium*, *Parasutterella*, *Hungatella*, and *Akkermansia* etc. enriched at week 10 (Supplementary Figure S4B).

### Correlation networks changed during *Helicobacter pylori* eradication

**Figure 4:**
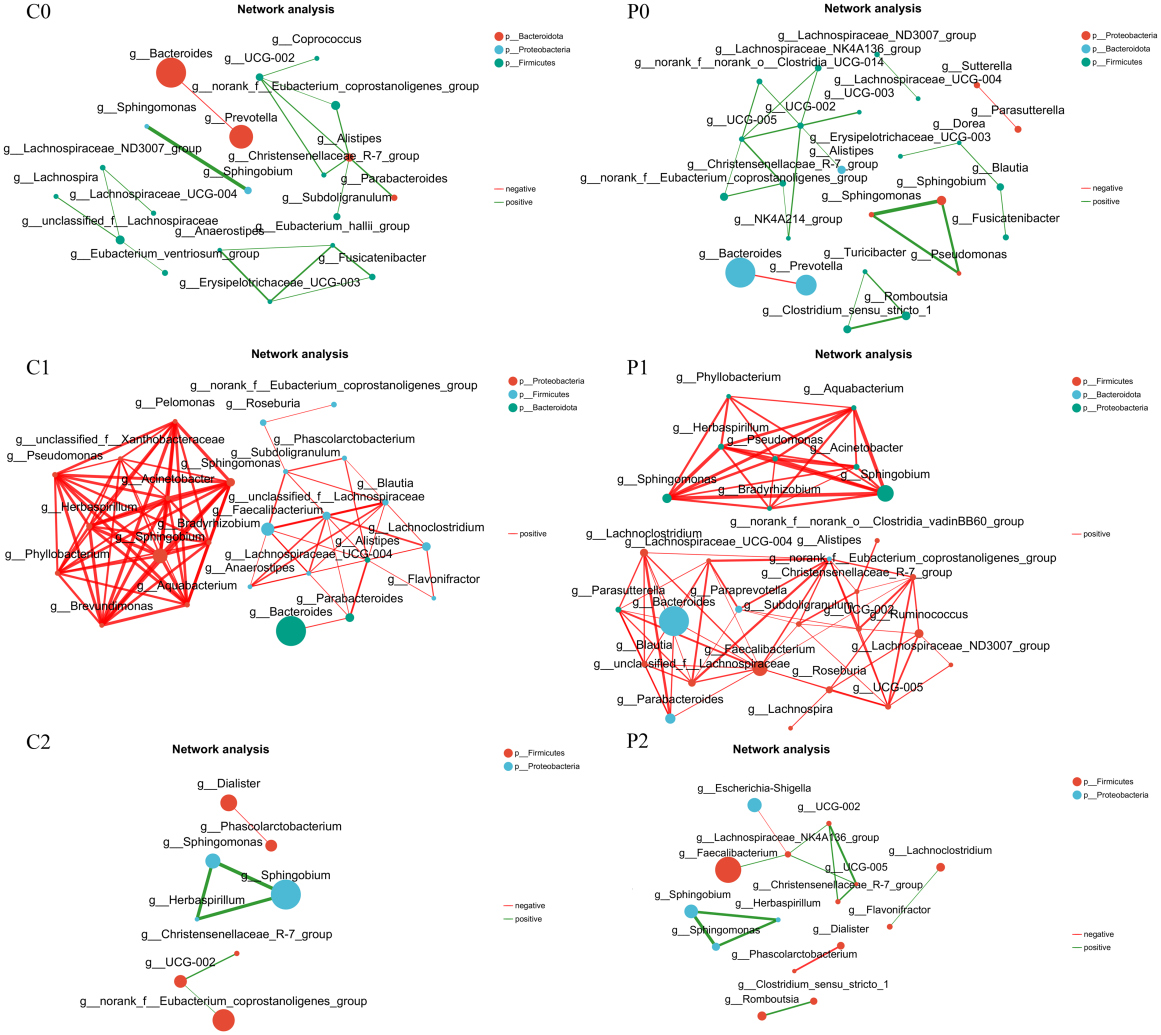
Correlation networks changed during *Helicobacter pylori* eradication. P, probiotic group; C, placebo group; P0 (*n* = 50)/C0 (*n* = 50), pretreatment; P1 (*n* = 50)/C1 (*n* = 50), cessation of quadruple therapy; P2 (*n* = 50)/C2 (*n* = 50), 8 weeks after quadruple therapy.

Correlation networks is useful tool for understanding the overall function and dynamic changes of microbial communities, which is of great significance for studying the role of microbial communities in health and disease states. In this study, the correlation networks of the gut microbiota were conducted in different group and time points. As shown in Figure 4, it was found that the correlation networks changed obviously during *H. pylori* eradication. Before *H. pylori* eradication, the correlation networks were simple in both groups and most connections were is negatively correlated. After *H. pylori* eradication, the correlation networks became complex in both group and all the connections were positively correlated. Of them, genera of *Faecalibacterium* and *Alistipes* were the keystone genus in the probiotic group at week 2. And the genera of *Phyllobacterium*, *Pseudomonas*, and *Sphingomonas* et al. were the keystone genus in the placebo group at week 2. At week 10, the correlation networks recovered to initial level and its complexity was even lower than initially in both groups. Of them, the correlation networks of the probiotic group seem more closer to the initial level.

## Discussion

Our study demonstrated that adjunctive probiotic therapy significantly mitigated the incidence of adverse events associated with *H. pylori* eradication and enhanced GSRS scores, but did not affect the eradication rate. Notably, the eradication of *H. pylori* was associated with transient alterations in the gut microbial ecology and structure. Probiotic supplementation was effective in counteracting antibiotic-induced disturbances in gut microbiota composition.

Numerous studies have explored the efficacy of probiotics as adjuncts in the treatment of *H. pylori* infection, but their clinical outcomes remain contentious (21–23). Our findings showed no statistically significant difference in the eradication rate between the two groups. Both regimens achieved high eradication rates, which may constrain the potential of probiotics to significantly affect these rates. Previous meta-analyses have suggested that the addition of probiotics enhances eradication rates primarily when the baseline rates in the control group are low (24). Our findings suggest that when the eradication protocol itself is highly effective, probiotics may play a limited role in enhancing the eradication rate and that improvements may be better sought through alternative strategies. In addition, this study was not an epidemiologic study focused solely on eradication rates, and the sample sizes in each group were relatively small. Therefore, comparisons of eradication rates require confirmation through further research.

Regarding adverse effects of *H. pylori* eradication, in this study, supplementation with probiotic preparations did not significantly reduce the overall incidence of adverse effects of *H. pylori* eradication owing to the limited sample size. However, probiotic-assisted bismuth quadruple therapy significantly reduced the incidence of nausea in patients, and there was a trend toward a reduction in the incidence of diarrhea. Besides, *B. animalis* subsp. *lactis* BLa80 was used as adjunct probiotic for the treatment of *H. pylori* and there are more microbes of genus *Bifidobacterium* at week 2 in the probiotic group. It is postulated that this may be related to the improvement of GSRS score.

Antibiotics are one of the main drugs used to eradicate *H. pylori* and have a significant effect on the gut microbiota (7, 11). Probiotics can improve the side effects of *H. pylori* eradication; however, there is little research on their impact on the gut microbiota of patients receiving *H. pylori* eradication treatment. It was reported that the *α*-diversity of microbiota of control group was still lower than that of initial level, as well as the *β*-diversity was significantly different from the initial 4 weeks after *H. pylori* eradication (22). Our results also found that the α-diversity decreased and β-diversity changed significantly after antibiotic intervention. After cessation of medication, under the intervention of maltodextrin and probiotic BLa80, the α-diversity of the gut microbiota recovered to initial level in both groups. However, the β-diversity of the probiotic group at week 10 was still different from that of day −1 (R = 0.0599, *p* = 0.001) and differed from the results of the control group. The genera of *Lachnoclostridium*, *Parasutterella*, *Hungatella*, and *Akkermansia* enriched in the probiotic group at week 10. *Akkermansia* is regarded as a next-generation probiotic that plays an important role in maintaining the integrity of the intestinal barrier, modulating the host immune response, and improving several metabolic pathways (25, 26). *Lachnoclostridium* is also considered beneficial for acetic acid production (27). *Hungatella* was reported to be associated with a decreased risk of narcolepsy type 1 (28). It has been postulated that it may have other beneficial effects. Therefore, enrichment of these beneficial microbes may have sustained positive effects in patients.

This study had several limitations. First, the sample size was insufficient and power analysis showed a relatively low power as 0.516. Future studies with larger sample sizes are needed. Second, samples were not collected from *H. pylori* negative individuals, therefore, it is difficult to determine whether eradication therapy restores the gastrointestinal microbiota to a composition like that of healthy individuals. Third, although this was a prospective study, a longer follow-up duration is necessary to determine the consequences of antibiotic-induced alterations in the gut microbiota, as samples were collected for only 2 months after treatment. Additionally, this study lacked the data on potential confounding factors, such as diet and other lifestyle-related variations, and data on antibiotic resistance of *H. pylori*, limiting the assessment of effect of probiotics on the eradication of resistant strains. Finally, fecal samples are not representative of the entire gut microbiota, as the gut microbiota differs depending on the location.

## Conclusion

Perturbation of the gut microbiota after *H. pylori* treatment appeared to be transient. *B. animalis* subsp. *lactis* BLa80 supplementation reduced the antibiotic-induced alterations in the gut microbiota and enriched some beneficial microbes in patients. Additionally, *B. animalis* subsp. *lactis* BLa80 combined with bismuth quadruple therapy significantly reduced the patients’ GSRS scores and the incidence of adverse events. This study provided evidence for the effectiveness of probiotic supplementation in improving gastrointestinal symptoms.

## Data Availability

The datasets presented in this study can be found in online repositories. The names of the repository/repositories and accession number(s) can be found in the article/Supplementary material.

## Footnotes

^1^ https://cloud.majorbio.com
