# Supplementary material for: Effect of probiotic supplementation combined with bismuth-containing quadruple therapy on gut microbiota during Helicobacter pylori eradication: a randomized, double-blind, placebo-controlled trial
Source: Front Nutr. 2024 Oct 16;11:1484646. doi: 10.3389/fnut.2024.1484646 (PMC11521887; doi:10.3389/fnut.2024.1484646)
Supplement: Supplementary file 1 [file Table_1.DOCX]

**Table S1. Frequencies of adverse treatment effects in each group**

| **Adverse effect, n (%)** | **Placebo group** | **Probiotic group** | | ***P* value** | |
| --- | --- | --- | --- | --- | --- |
|  | (n = 50) | (n = 50) | |  |  |
| Dysgeusia (metallic taste) | 26.00% (13/50) | 24.00% (12/50) | | 0.817 | |
| Nausea | 24.00% (12/50) | 8.00% (4/50) | | 0.029 | |
| Diarrhea | 14.00% (7/50) | 2.00% (1/50) | | 0.065 | |
| Vomiting | 6.00% (3/50) | 6.00% (3/50) | | 1.000 | |
| Abdominal pain | 2.00% (1/50) | 2.00% (1/50) | | 1.000 | |
| Bloating | 8.00% (4/50) | 4.00% (2/50) | | 0.674 | |
| Constipation | 10.00% (5/50) | 6.00% (3/50) | | 0.712 | |
| Rash | 0.00% (0/50) | 2.00% (1/50) | | 1.000 | |
| Lethargy | 4.00% (2/50) | 0.00% (0/50) | | 0.475 | |
| Total | 46.00% (23/50) | 36.00% (18/50) | | 0.309 | |
|  |  |  |  | |  |
