# Supplementary material for: Effect of probiotic supplementation combined with bismuth-containing quadruple therapy on gut microbiota during Helicobacter pylori eradication: a randomized, double-blind, placebo-controlled trial
Source: Front Nutr. 2024 Oct 16;11:1484646. doi: 10.3389/fnut.2024.1484646 (PMC11521887; doi:10.3389/fnut.2024.1484646)
Supplement: Supplementary file 2 [file Table_2.DOCX]

**Table S2. Pre-, post-treatment GSRS scores and mean difference within each group**

| **GSRS subscale** | **Placebo group^a^** | ***P* value** | **Probiotic group^a^** | ***P* value** |
| --- | --- | --- | --- | --- |
|  | (n = 50) |  | (n = 50) |  |
| Pre-treatment (week 0) | 6.64 (1.75-9.25) | ＜ 0.001^b^ | 6.96 (2.00-9.25) | ＜ 0.001^b^ |
| Post-treatment (week 2) | 3.34 (0.00-5.00) |  | 1.30 (0.00-2.00) |  |
| Mean difference | 3.30 (0.00-8.00) |  | 5.66 (1.00-9.00) | 0.041^c^ |

GSRS, Gastrointestinal Symptom Rating Scale.

^a^ Data were presented as median and interquartile range.

^b^ Comparisons between pre- and post-treatment GSRS scores within each group.

^c^ Comparisons of mean difference of GSRS score between the placebo and probiotic group.
