# Supplementary figures and images for: Effect of probiotic supplementation combined with bismuth-containing quadruple therapy on gut microbiota during Helicobacter pylori eradication: a randomized, double-blind, placebo-controlled trial
Source: Front Nutr. 2024 Oct 16;11:1484646. doi: 10.3389/fnut.2024.1484646 (PMC11521887; doi:10.3389/fnut.2024.1484646)

**Figure S1. Mean difference in GSRS score between groups.**

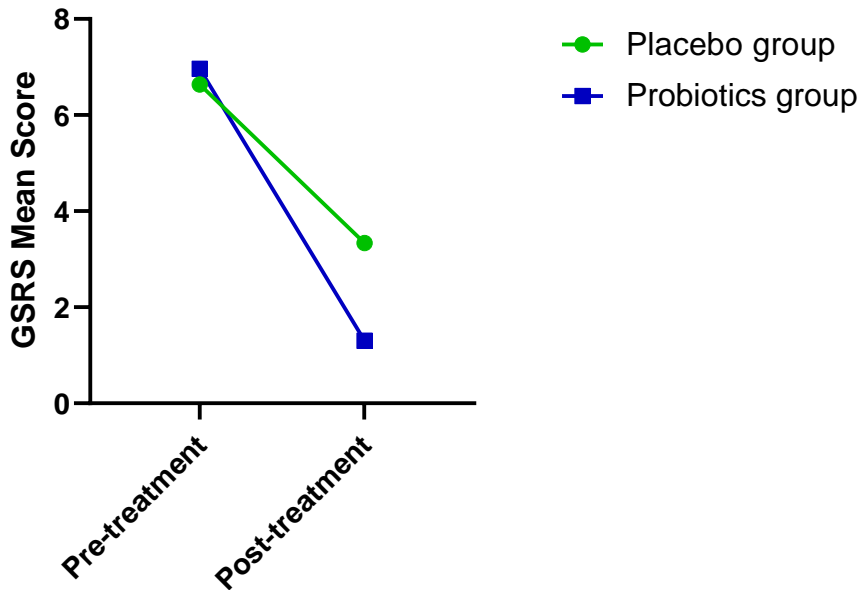

Supplement: Supplementary file 3 [file Image_1.pdf]

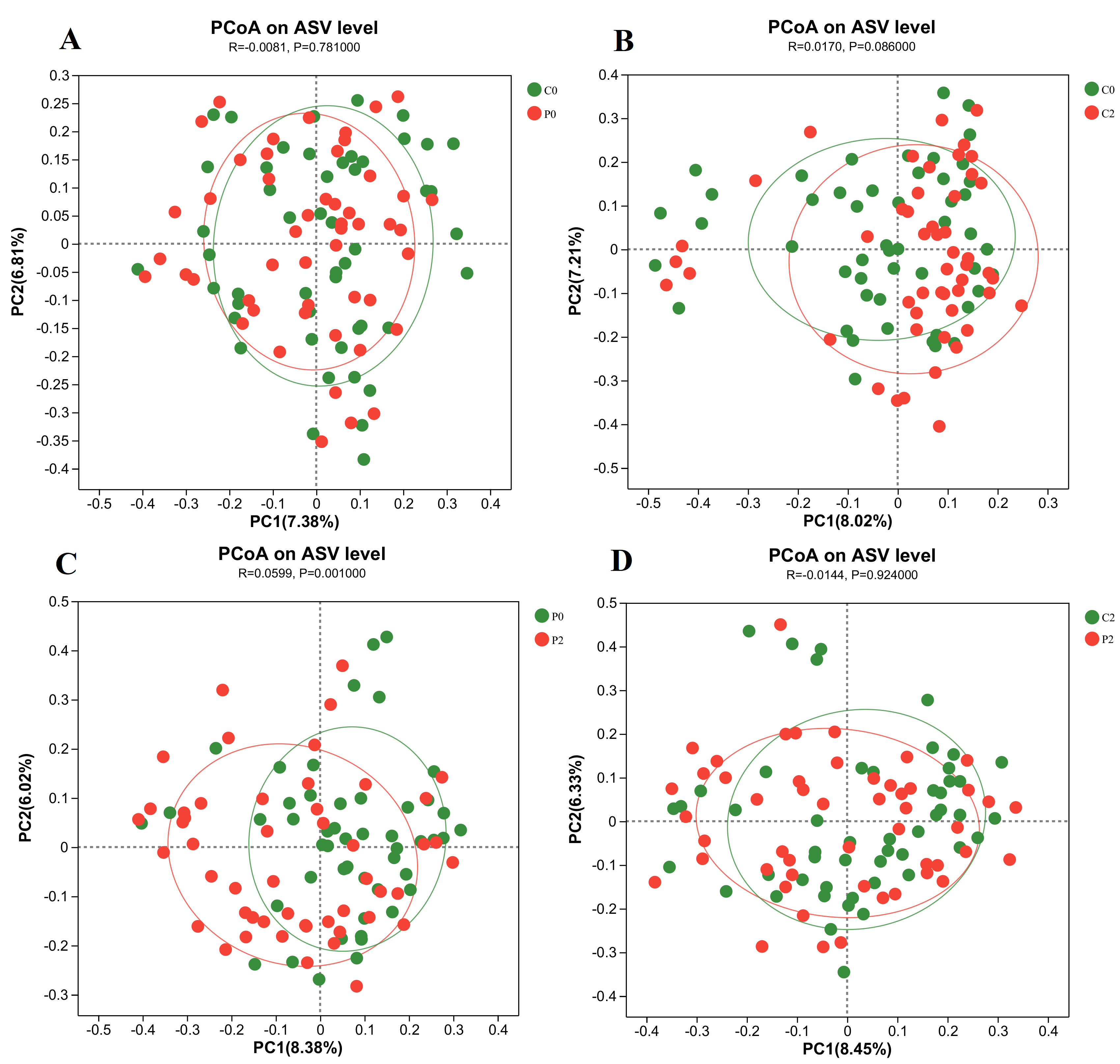

Supplement: Supplementary file 4 [file Image_2.tif]

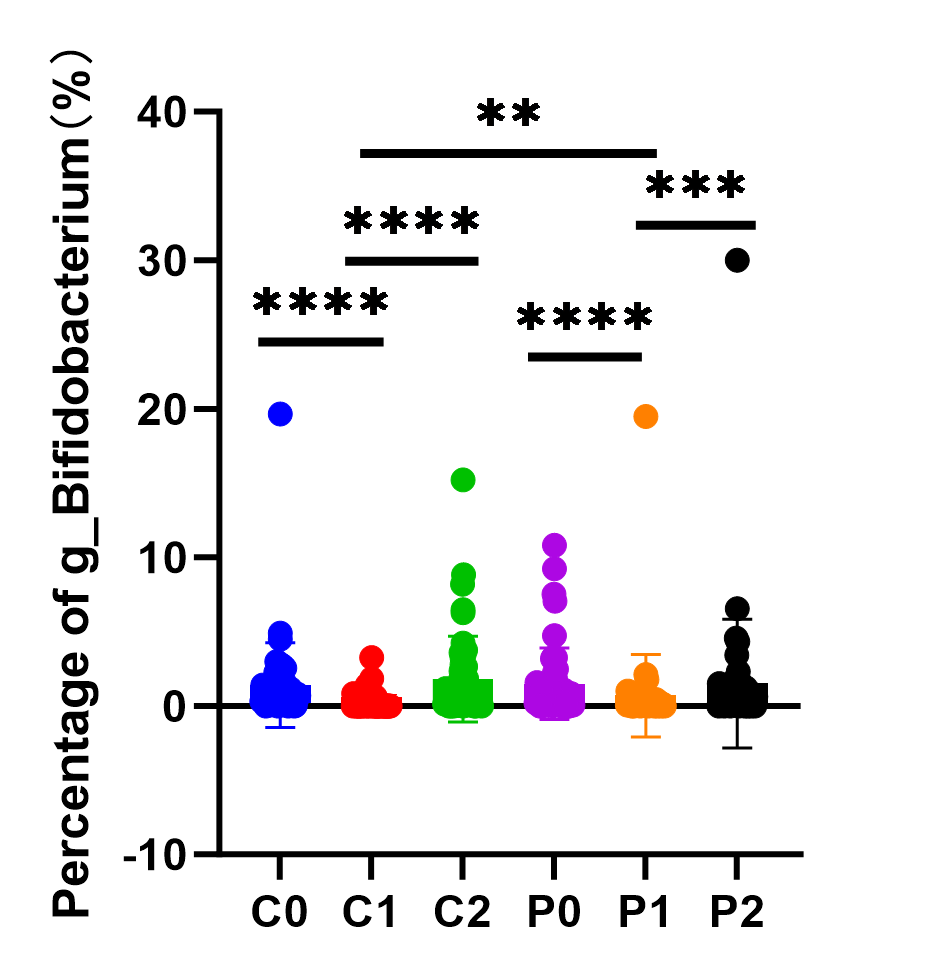

Supplement: Supplementary file 5 [file Image_3.tif]

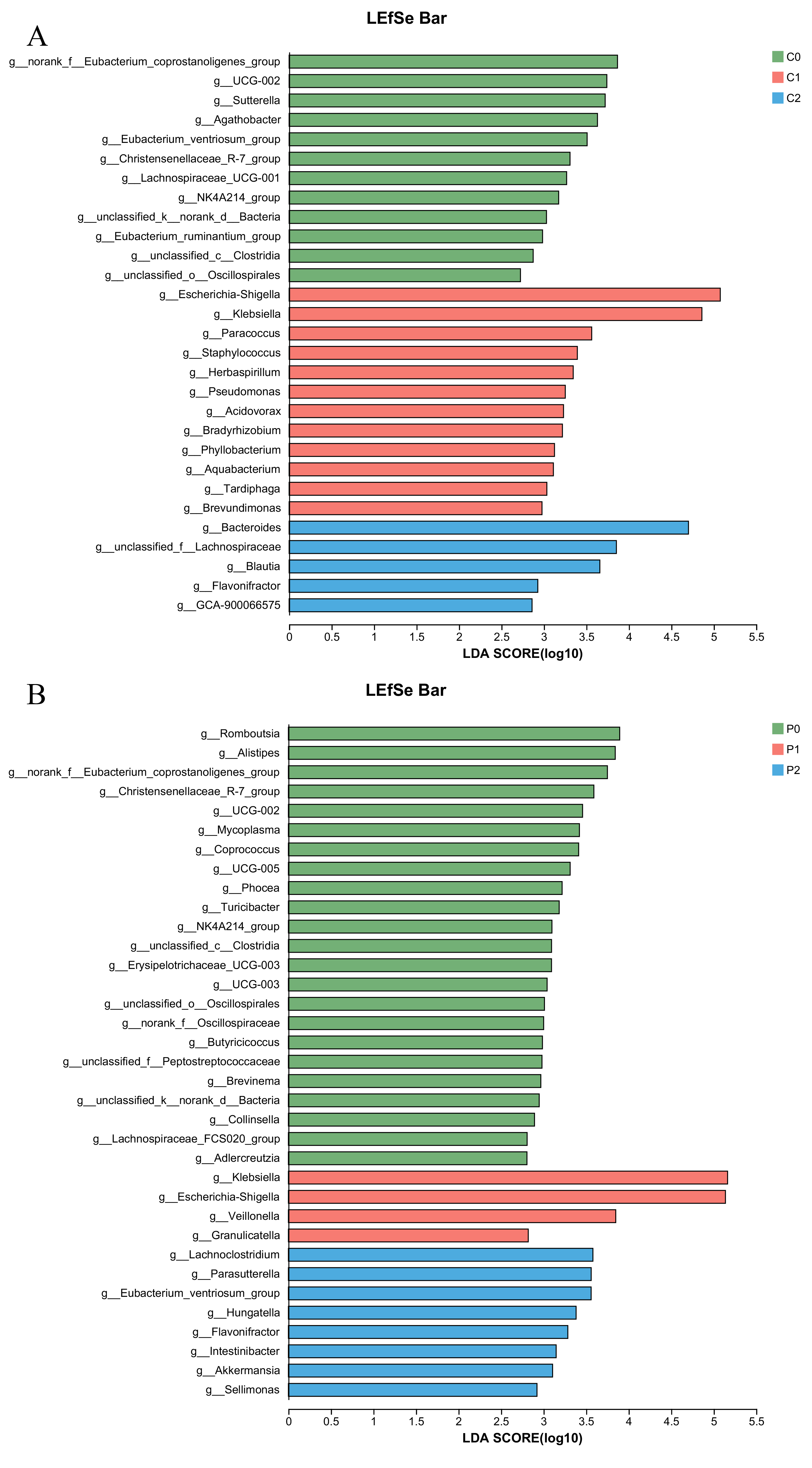

Supplement: Supplementary file 6 [file Image_4.tif]
